# Supporting long‐term sustainability of ex situ collections using a pedigree‐based population management approach

**DOI:** 10.1002/aps3.11491

**Published:** 2022-09-26

**Authors:** Jeremy A. Foster, Seana K. Walsh, Kayri Havens, Andrea T. Kramer, Jeremie B. Fant

**Affiliations:** ^1^ Program in Plant Biology and Conservation Northwestern University 2205 Tech Drive Evanston Illinois 60208 USA; ^2^ Negaunee Institute for Plant Conservation Science and Action, Chicago Botanic Garden 1000 Lake Cook Road Glencoe Illinois 60022 USA; ^3^ Department of Science and Conservation National Tropical Botanical Garden 3530 Papalina Road Kalāheo Hawaiʻi 96741 USA; ^4^ Natural History Museum of Denmark University of Copenhagen DK‐2100 Copenhagen Denmark

**Keywords:** *Brighamia insignis*, genetic diversity, inbreeding, pedigree, pollen viability, strategic crosses

## Abstract

**Premise:**

Living collections maintained for generations are at risk of diversity loss, inbreeding, and adaptation to cultivation. To address these concerns, the zoo community uses pedigrees to track individuals and implement crosses that maximize founder contributions and minimize inbreeding. Using a pedigree management approach, we demonstrate how conducting strategic crosses can minimize genetic issues that have arisen under current practices.

**Methods:**

We performed crosses between collections and compared progeny fitness, including plant performance and reproductive health. We genotyped the progeny and parental accessions to measure changes in diversity and relatedness within and between accessions.

**Results:**

The mean relatedness values among individuals within each accession suggest they are full siblings, demonstrating that there was high inbreeding and low diversity within accessions, although less so among accessions. Progeny from the wider crosses had increased genetic diversity and were larger and more fertile, while self‐pollinated accessions were smaller and less fertile.

**Discussion:**

Institutions that hold exceptional species should consider how diversity is maintained within their collections. Implementing a pedigree‐based approach to managing plant reproduction ex situ will slow the inevitable loss of genetic diversity and, in turn, result in healthier collections.

For threatened plant species, ex situ collections are a crucial component of conservation plans aimed at minimizing extinction risk (Pritchard et al., 2012; Krishnan and Novy, 2016). However, for these collections to have maximum conservation value (i.e., for potential future use in population augmentations and/or restoration in situ; Laliberté, 1997; Mounce et al., 2017), their genetic diversity must be representative of in situ populations (Hoban et al., 2020) and sustainably maintained for the lifetime of the collection (Fant et al., 2016). Multiple recommendations exist on the optimal sampling to maximize diversity of a conservation collection (Marshall and Brown, 1975; Guerrant et al., 2004; Hoban and Schlarbaum, 2014). For species with orthodox seeds (i.e., those that can be dried and stored at low temperatures for many years), traditional seed banking enables conservation practitioners to maintain the most genetic diversity long term at the lowest cost (Kramer et al., 2011). For exceptional plant species (i.e., those with seeds that do not remain viable when stored for long periods of time using traditional seed banking methods), most ex situ conservation is achieved via living collections (Wood et al., 2020). Maintaining living plant collections presents many challenges not faced by seed banking, including an increased risk of unintentional hybridizing with closely related taxa in the greenhouse or garden grounds, inbreeding with closely related individuals, and adaptation to cultivated growing conditions (Havens et al., 2004; Wood et al., 2020), which may result in changes to, or loss of, genetic diversity in the collection.

Maintaining sufficient numbers of living plants to maximize diversity and minimize inbreeding and losses due to genetic drift requires significantly more resources relative to seed banking, and often more than can be achieved by a single institution (Fant et al., 2016; Wood et al., 2020). Hence the optimal approach is to develop multiple collections managed across institutions (known as metacollections; Griffith et al., 2019). This approach has numerous advantages including building redundancy, which safeguards against loss due to changes in institutional priorities and/or stochastic events, and minimizing any net loss of diversity due to genetic drift. Through exchanges between institutions, potential issues associated with inbreeding and adaptation to local horticultural conditions can be minimized as well. The transfer of material between institutions is particularly critical if collections are maintained for multiple generations, given that most institutions will maintain only a few plants from a limited number of sources. Optimally maintaining genetic diversity within a metacollection requires carefully documenting and tracking material and its associated provenance data, and maintaining the genetics of founders by employing strategic crosses (St. Clair et al., 2020; Wood et al., 2020; Gavin‐Smyth et al., 2021).

The challenges faced by botanic gardens to manage genetic diversity in metacollections of exceptional plant species closely parallel those that were faced by the zoological community in maintaining captive animal populations within and across institutions (Lacy, 1987; Frankham, 2008). Zoos addressed these challenges through the creation and adoption of a shared studbook database and software program to manage metapopulations of animals. The studbook database (Species360 Zoological Information Management System [ZIMS], 2021) holds a pedigree of all individuals that can be traced back to founders and has features that allow curators to easily track movement of animals or gametes within and between institutions. A pedigree management software (PMx) provides the necessary information that managers need to make decisions to assist with the demographic and genetic management of animal populations (Lacy et al., 2012). Some barriers to using a pedigree approach for plant management have already been identified (see Fant et al., 2016; Wood et al., 2020), but one major issue for genetic management is the lack of standards associated with assignments of accessions. Across botanical institutions, and even within the same institution, an accession can represent a collection from a single individual, a maternal line, or even a single source population. Each of these accession types differ in the genetic relationship between individuals within and among accessions, and therefore differ in how management decisions would be implemented. In order to help overcome the challenges of maintaining exceptional plant species ex situ, a studbook database for plants is currently being developed and PMx is being adapted for use with plants (as PMxceptional) using *Brighamia insignis* A. Gray (Campanulaceae) as a case study (Wood et al., 2020).

*Brighamia insignis* has become a model for exceptional species collections management (Walsh, 2015; Fant et al., 2016; Griffith et al., 2020; Wood et al., 2020). Seed freeze sensitivity is observed at the family level, and preliminary data at the species level suggest that seeds survive storage at 5°C for only 5–10 years and at −18°C for an even shorter period (Chau et al., 2019). For this reason, the species is classified as exceptional under exceptionality factor 3 (EF3; Pence et al., 2022). A recent seed dormancy and germination study of the only other species in the genus, *B. rockii* H. St. John, found that seeds are able to germinate in darkness, suggesting the species is unlikely to form a long‐lived soil seed bank (Wolkis et al., 2022). If we assume *B. insignis* seeds behave similarly, these findings further support the notion that reintroductions will be needed to reestablish extirpated populations. Collections of *B. insignis* have been maintained primarily as live plants in at least 52 ex situ sites currently worldwide (Botanic Gardens Conservation International, 2021). The broad ex situ distribution of *B. insignis* across multiple botanical institutions and limited tracking have led to genetic concerns overall for the metacollection of the species (Walsh, 2015; Fant et al., 2016; Wood et al., 2020); these concerns are supported by an observed decline in plant health, including pollen production and viability, in some collections. Walsh et al. (2019) found that the majority of plants in the primary collection at the National Tropical Botanical Garden (NTBG) had low pollen production and viability as early as 2013 and suspected that one of the reasons could be due to inbreeding. With many collections of the species being perpetuated through selfing or crossing with closely related individuals within the same institutional collection (e.g., same accession), it is suspected that collections of *B. insignis* have become inbred.

To address the concerns of genetic decline in collections of exceptional plant species, we performed strategic crosses between accessions of *B. insignis* to test the hypothesis that employing a broader pedigree approach to crosses will increase genetic diversity and decrease inbreeding in collections, which will in turn increase vigor in progeny and improve collection health. The progeny were genotyped using microsatellite markers to compare different measures of genetic diversity and inbreeding. Measures of plant performance, including plant height, pollen production, and pollen viability, were compared among the different progeny. We demonstrate that adapting the pedigree management approach developed by zoos can overcome the genetic challenges of metacollection management for exceptional plant species.

## METHODS

### Study species

*Brighamia insignis* is a caudiciform succulent that is historically endemic to the westernmost main Hawaiian Islands of Kaua‘i and Ni‘ihau, where it occurred on sea cliffs between sea level and 400 m elevation (Lammers, 1989; Wagner et al., 1999). The species has been listed as Endangered by the United States Fish and Wildlife Service since 1994 (USFWS, 2021) and is assessed on the International Union for the Conservation of Nature (IUCN) Red List of Threatened Species as Critically Endangered (Possibly Extinct in the Wild) (Walsh, 2016). *Brighamia insignis* was pronounced extinct in the wild in 2020 after numerous drone surveys confirmed the last known individual on the Nā Pali Coast of Kaua‘i was no longer present (K. Wood, National Tropical Botanic Garden, personal communication).

### Collections

Over 250 *B. insignis* plants are maintained in ex situ collections at over 52 institutions globally (Botanic Gardens Conservation International, 2021). Most institutions maintain only a single accession, which usually represents a single seed source. If more than one accession is maintained, they are usually represented by progeny from multiple generations of crosses (e.g., F_1_ and F_2_ generations). The exception is the collection curated by NTBG on Kaua‘i, which is the largest, primary collection. Some accessions represent original, wild collections while others represent up to five generations of crosses. Some accessions, including ones representing distinct wild founders, have been lost, and many of the original and oldest accessions are reduced to only one or two representatives (Wood et al., 2020). As the original collections were made by botanists from NTBG starting in the 1970s, it is assumed that all other collections originate from the NTBG collection. Hence, the accessions from the United States Botanic Garden (USBG), San Diego Zoo (SDZ), and Chicago Botanic Garden (CBG) used in this study all likely originated from one of the original NTBG collections, although the original provenance is lost in these and almost all cases, and many of the accessions have gone through two or three generations since the original transfer.

### Crosses

We generated five new accessions of *B. insignis* at CBG that represent five cross types, including a self‐pollinated accession within CBG's original collection and four pollen donors represented by collections curated at three different institutions. One of the pollen donors was an individual derived from one of the wild‐sourced accessions at NTBG; this accession is one of 23 accessions that were collected in the 1990s. The remainder were from SDZ and two accessions from USBG (USBG1 and USBG2), which are likely siblings. Pollen from plants held at the different institutions was collected by their representative curators and mailed to CBG to cross with a maternal plant in the CBG collection. Once received, pollen was applied directly to receptive stigmas, and flowers were tagged to indicate the different crosses. For the self‐pollination treatment, pollen was collected from a separate flower and applied to the receptive stigma of a flower on the same plant. We performed a geitonogamous pollination as opposed to a same‐flower pollination as *B. insignis* is protandrous and primarily outcrossing (Walsh et al., 2019). Furthermore, the purpose of this study was not to explore the breeding system but rather to compare the fitness of progeny resulting from self‐pollination and the different crosses. Fruit was collected once mature (splitting of capsule), and the seeds were extracted. Seeds representing the five different accessions were all sown on the same day in 2016, thus plants are all the same age. As the maternal founder is the same for all crosses, the different accessions represent half siblings. All plant performance measurements were taken in October 2020.

### Genetic measures

Genomic DNA was extracted from the parental accessions using the 2× cetyltrimethylammonium bromide (CTAB) method (Doyle and Doyle, 1987) and from the progeny of the aforementioned crosses using the DNeasy Plant Mini Kit (catalog no. 69104, Qiagen, Germantown, Maryland, USA). For the parental accession plants, we extracted DNA from the maternal accession (CBG) and from three of the four paternal accessions (SDZ [accession 2001‐0273‐022], NTBG [accession 990833.3], and USBG1 [accession 2014‐0070]). As the fourth accession (USBG2) died before we could extract DNA, we used progeny derived from a self of that plant (accession 2019‐000) to estimate paternal contribution. All samples were genotyped using microsatellite markers and protocols described in Fant et al. (2019), which had already been tested for Hardy–Weinberg equilibrium and presence of null alleles. From this previous work, we identified a total of 11 primers that were reliable and polymorphic that would be useful for this study. They included eight primers (B05, B08, B43, B44, B46, B47, B51, B57) that were designed for *B. insignis* (Fant et al., 2019) and three primers (L23, L33, L34) that were originally designed for *Lobelia villosa* (Rock) H. St. John & Hosaka, which is not surprising as both species are part of the Hawaiian lobeliad clade (Barbará et al., 2007). The eight *B. insignis* primers were visualized using pre‐labeled forward primers with either WellRed Black (D2), Green (D3), or Blue (D4) fluorescent dye (Sigma‐Aldrich, St. Louis, Missouri, USA), while for *L. villosa* markers, the forward primer was modified at the 5′ end (5′‐CACGACGTTGTAAAACGAC‐3′) so they could be labeled separately (Schuelke, 2000). All products were analyzed and scored using a CEQ 8000 Genetic Analysis System V9.0 (Beckman Coulter, Brea, California, USA). Given that *B. insignis* is a paleotetraploid (Lammers, 1988), four of the primer pairs (B44, B47, B51, and L23) produced more than two bands. As the peaks were separated from other alleles by a large range (20–30 bp) and segregated independently (Fant et al., 2019), they were scored as independent loci.

### Fitness

To compare fitness components among progeny of the different cross types, we measured plant height in centimeters, stem diameter in centimeters, number of inflorescences, pollen production in milligrams, and pollen viability for five to nine plants per accession. Plant height was measured from the soil surface to the apical meristem using a measuring tape. Plant diameter was measured at the widest point of the caudex using calipers. To determine pollen production per accession, we obtained an average of pollen mass per flower. Ten flowers were collected from each accession at anthesis, and the pollen was collected by scraping it out of the anther column using a probe. The anther column was then placed into a 1.5‐mL microcentrifuge tube and vortexed to help release all of the remaining pollen. All the pollen from each flower was then placed into a separate pre‐weighed 1.5‐mL microcentrifuge tube and weighed within 1 h of collection using an analytical balance readable to within 0.1 mg (A&D HR‐200; A&D Weighing, Ann Arbor, Michigan, USA). Average pollen mass per flower for an accession was calculated by dividing the combined mass of the 10 sampled flowers by 10.

To assess pollen viability, pollen was collected at anthesis from three to five flowers from each plant that was flowering. Using a dental curette, pollen was scraped from the connate anthers directly into a 1.5‐mL microcentrifuge tube. Pollen from all plants belonging to the same accession was aggregated and viability was assessed within 1 h of being collected using a *p*‐phenylenediamine stain, which detects non‐viable pollen (Rodriguez‐Riano and Dafni, 2000). The freshly prepared stock stain solution was kept in the dark at 4°C to avoid precipitation and prevent it from turning dark, then heated to 37°C using a water bath to activate. Approximately 300 pollen grains per replicate for three replicates for each accession were placed on a slide and stained with two drops of the activated stain, and the pollen cells were then counted under a compound microscope (Leica DMLB100T Type 020‐519.511; Leica Microsystems, Wetzlar, Germany). Pollen that was round and stained dark brown or black was considered viable, whereas shriveled pollen with a very faint stain or no stain was considered dead (Rodriguez‐Riano and Dafni, 2000). Mean percentage pollen viability per accession was subsequently calculated by dividing the number of viable pollen grains by the 300 total pollen grains counted per accession.

### Data analysis

Using the 15 scored loci, we calculated genetic diversity using expected heterozygosity (*H*_e_), percent polymorphic loci, and the number of private alleles, which are less sensitive to small sample sizes, using GenAlEx (Peakall and Smouse, 2006). Weir and Cockerham's (1984) estimates of Wright's inbreeding coefficient (*F*_IS_) and the Lynch–Ritland mean estimator of relatedness (*r*) (Lynch and Ritland, 1999) were also calculated in GenAlEx to compare inbreeding within accessions.

Analysis of variance (ANOVA) and post‐hoc Tukey's honest significant difference (HSD) tests were used to compare differences in plant height, stem diameter, number of inflorescences, pollen production, and pollen viability among progeny resulting from the different crosses. Analyses were completed in R version 4.0.5 (R Core Team, 2021) and RStudio version 1.4.1106 (RStudio Team, 2021) with the packages tidyverse (Wickham et al., 2019), ggplot2 (Wickham, 2016), knitr (Xie, 2021), and gridExtra (Auguie and Antonov, 2017). Significance was accepted at an alpha (α) level of 0.05. All means are presented ± one standard deviation (SD).

## RESULTS

### Genetic comparison of parental accessions

Genetic diversity and number of polymorphic loci were lowest for the accessions at USBG (USBG1 and USBG2) and CBG, followed by NTBG, and highest for the accession at SDZ (Table 1). The same trend was seen in the number of unique alleles, with the accession at SDZ having the highest number of alleles not found in other accessions, followed by the accession at NTBG. The higher diversity in the SDZ accession, which was even higher than in the wild‐collected accession from NTBG, would suggest that this accession might have been derived from a distant outcross between unrelated individuals; however, unfortunately we do not have any information on the cross that generated this accession. The relatedness values revealed that all plants within each accession were likely full siblings (*r* ~ 0.5). This is consistent with accession tracking by maternal line, although this also suggests that there is only a single paternal donor. Interestingly, inbreeding levels within the collections varied. Accessions USBG2 (there were insufficient data to calculate this for USBG1 accession) and SDZ had very high inbreeding levels. The two accessions from USBG were also closely related to each other (*r* = 0.37; Table 1), confirming that those accessions are also siblings. By contrast, the accessions from all four institutions were unrelated to each other (*r* ≤ 0; Table 1).

**Table 1:** Measures of genetic diversity of accessions from the Chicago Botanic Garden (CBG), National Tropical Botanical Garden (NTBG), San Diego Zoo (SDZ), and United States Botanic Garden (USBG1&2) that were used as pollen donors in this study.

Institution	Accessions	Indv. (*N*)	Diversity^a^			Inbreeding^b^		Divergence (Relatedness of collections to each other)			
			*H*_e_	Poly loci	Prv	*F*	*r*	CBG	NTBG	USBG1	USBG2
CBG	Various	9	0.07	20%	0	−0.17	0.48				
NTBG	990833	14	0.10	47%	6	−0.05	0.40	−0.11			
USBG1	2014‐0070	2	0.09	20%	0	*	0.50	−0.27	−0.32		
USBG2	2019	30	0.06	27%	1	0.36	0.45	−0.27	−0.27	0.37	
SDZ	2001‐0273	6	0.41	80%	15	0.56	0.50	0.01	0.03	−0.28	−0.24

*Note*: *N* = number of samples.

*Insufficient data to calculate value.

^a^ Genetic diversity is measured using expected heterozygosity (*H*_e_), percent polymorphic loci (Poly loci), and number of private alleles (Prv).

^b^ Inbreeding is calculated using the Weir and Cockerham (1984) inbreeding coefficient (*F*) and relatedness (*r*) uses the Lynch and Ritland (1999) mean estimator.

### Genetic comparison of progeny

The progeny resulting from the crosses between the CBG plants and the four paternal accessions showed an increase in genetic diversity and polymorphic loci in three of the five crosses (SDZ, USBG1, and USBG2) compared to the progeny resulting from the NTBG cross and the self treatment. For these crosses, there was a threefold increase in genetic diversity and percent polymorphic loci compared to the original maternal plant (CBG; Table 2). There was also an increase in genetic diversity in the progeny that resulted from crosses with USBG paternal plants compared to the original USBG accessions. However, there was a decrease in genetic diversity and polymorphic loci in progeny from the SDZ paternal cross compared to the original SDZ accessions. The two crosses that showed no increases in genetic diversity or percent polymorphic loci were the self treatment and the NTBG cross (Tables 1 and 2). The high average relatedness values (0.16–0.47; Table 2) of offspring to their parental genotypes are consistent with expectations for any offspring–parental relationship, confirming most of our crosses were successful. The exception was the CBG × NTBG cross, for which the relatedness values of the progeny to the parental accessions suggest they are only closely related to the CBG accession (*r* = 0.47), and not related to any NTBG individuals (*r* = −0.14; Table 2). Given the low pollen produced by this accession, it is likely that these progeny are the product of self‐pollination rather than outcrossing. The genetic diversity of progeny from these crosses either remained the same or declined. The overall level of inbreeding in these progeny was consistent with expectations for each cross type, with moderately high inbreeding in selfed progeny (*F* = 0.12) and NTBG (*F* = 0.16) crosses, and negative inbreeding coefficients in the cross from SDZ (*F* = −0.63), USBG1 (*F* = −0.85), and USBG2 (*F* = −0.67) paternal plants, which is consistent with assortative mating.

**Table 2:** Measures of genetic diversity of progeny from controlled crosses and the relatedness to the paternal accessions from the Chicago Botanic Garden (CBG), National Tropical Botanical Garden (NTBG), San Diego Zoo (SDZ), and United States Botanic Garden (USBG1&2).

		Diversity^a^		Inbreeding	Relatedness				
Cross	Indv. (*N*)	*H*_e_	Poly loci	*F*_IS_	CBG	NTBG	USBG1	USBG2	SDZ
CBG × CBG	5	0.08	20%	0.24	0.39	—	—	—	—
CBG × NTBG^b^	4	0.04	13%	0.16	0.47	−0.14	—	—	—
CBG × USBG1	6	0.28	54%	−0.64^c^	0.28	—	0.25	0.16	—
CBG × USBG2	7	0.27	60%	−0.8^c^	0.39	—	0.21	0.25	—
CBG × SDZ	9	0.31	60%	−0.67^c^	0.17	—	—	—	0.29

*Note*: *N* = number of samples.

^a^ Genetic diversity is measured using expected heterozygosity (*H*_e_); percent polymorphic loci (Poly loci) and relatedness use the Lynch and Ritland (1999) mean estimator.

^b^ The genetic data suggest the progeny from this cross is likely self‐pollinated and not the result of cross with the intended NTBG accession.

^c^ The negative inbreeding coefficient (*F*_IS_) suggests that these crosses are the product of distant crosses.

### Fitness

No significant difference was found among progeny from the different crosses for the number of inflorescences per plant (*P* = 0.052), while differences in plant height and diameter were significant (*P* < 0.001). Plant height and diameter (in centimeters) were significantly higher in progeny derived from the SDZ cross (height = 51 ± 1.7, diameter = 131.5 ± 7.6), compared to progeny from the other three crosses (NTBG, USBG1, USBG2) and the self (*P* < 0.001 for all post‐hoc, pairwise comparisons). When measuring the average pollen production per flower (in milligrams), we found that plants from the self‐pollination treatment produced no measurable pollen. Among the progeny that produced pollen, pollen mass was significantly different (*P* < 0.001). The NTBG cross (likely self) produced the lowest pollen mass, while the SDZ progeny produced the highest. The differences in pollen mass between progeny were significant for all pairwise comparisons except for the two USBG crosses (*P* < 0.03).

**Figure 1:**
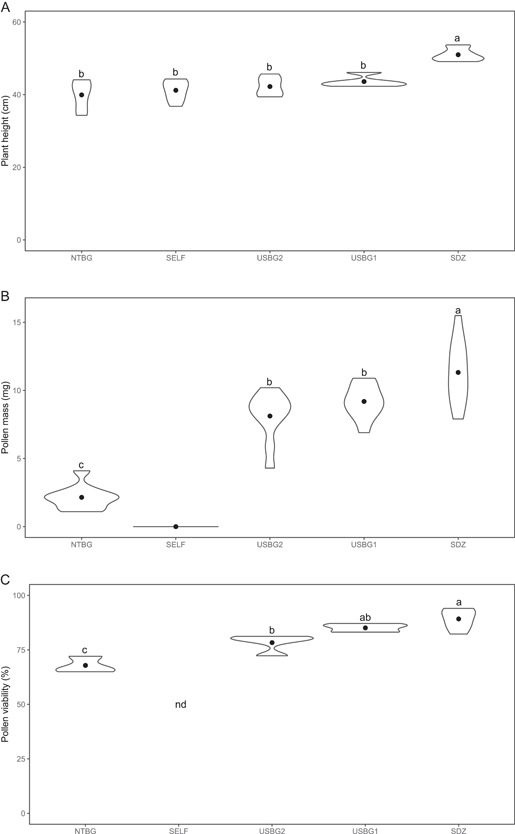
Plant height (A), pollen mass (B), and pollen viability (C) of *Brighamia insignis* progeny from a self‐pollination (SELF) and crosses using four different pollen donors (National Tropical Botanical Garden [NTBG], United States Botanic Garden [USBG2, USBG1], and San Diego Zoo [SDZ]) from three different institutions and a maternal plant at the Chicago Botanic Garden. Progeny from the different crosses are ordered by ascending genetic diversity (*H*_e_). The violin plot shows the distribution of numeric data around the mean. The point represents the median, and the wider areas represent a higher density of that variable. nd = no data (i.e., no pollen was produced by SELF progeny plants from which to assess pollen viability).

Similarly, there was a significant difference in pollen viability among progeny (*P* < 0.001). Pollen viability was lowest in the progeny resulting from the NTBG cross (67.88 ± 3.72%), which was significantly lower than progeny from the other crosses (*P* < 0.02 for all post‐hoc, pairwise comparisons). The highest percent pollen viability was in the progeny from the SDZ cross (89.22 ± 4.62%), although there was no significant difference in pollen viability between the SDZ and USBG1 (85.10 ± 1.82%) progeny (*P* = 0.31) or between the USBG2 (78.33 ± 4.08%) and USBG1 progeny (*P* = 0.07) (Figure 1, Table 3).

**Table 3:** Fitness data of the five accessions at the Chicago Botanic Garden resulting from controlled crosses.

Cross	Indv. (*N*)	Height (cm)	Base (cm)	Inflor. (count)	Pollen/flower (mg)	Pollen viability (%)
CBG × CBG	7	41.2 ± 2.6	116.2 ± 4.2	20.4 ± 2.8	0	—
CBG × NTBG	6	39.9 ± 4.1	110.5 ± 3.3	12.8 ± 1.5	2.15 ± 0.9	67.9 ± 3.72
CBG × USBG1	5	43.6 ± 1.5	112.6 ± 5.4	18.2 ± 2.6	9.2 ± 1.2	85.1 ± 1.82
CBG × USBG2	6	42.2 ± 2.6	115.2 ± 3.7	17.5 ± 1.2	8.1 ± 1.8	78.3 ± 4.08
CBG × SDZ	9	51.0 ± 1.7	131.5 ± 7.6	18.9 ± 6.6	11.3 ± 2.3	89.2 ± 4.62
Statistics		*F*_4,42_ = 33.6; *P* < 0.001	*F*_4,42_ = 6.67; *P* < 0.001	*F*_4,27_ = 2.70; *P* < 0.052	*F*_4,42_ = 69.01; *P* < 0.001	*F*_3,14_ = 23.93; *P* < 0.001

*Note*: *N* = number of samples.

## DISCUSSION

Maintaining the genetic integrity of any living plant collection comes with many challenges. Many of these can be overcome by adapting some of the same protocols and tools that zoos developed to manage metapopulations of animals. Here, we demonstrated that cross types affect levels of inbreeding and genetic diversity in progeny, which has repercussions on the fitness of new accessions that replace aging accessions in ex situ conservation collections. This is particularly critical for the extinct‐in‐the‐wild, exceptional species *B. insignis*, where higher levels of inbreeding resulted in smaller plants and decreased pollen production and viability, which could result in the complete loss of a founder or maternal line maintained ex situ. Given these results, we recommend the adoption of the zoo's pedigree‐based approach, focused on equalizing founder contribution and minimizing inbreeding, when propagating new plants to replenish aging accessions of exceptional species. Adapting this approach will help maintain the genetic integrity and health of collections. In practice, this will necessitate new protocols, for example, institutions must coordinate to share pollen and/or seeds in order to conduct strategic crosses between different accessions of plants, and refrain from opportunistically collecting spontaneously formed fruit (which would likely be the result of self‐pollination) to replenish aging living plant collections.

In this study, the progeny derived from a cross between individuals with low relatedness showed the greatest difference in the number of shared alleles and produced the most vigorous plants. The plants were significantly taller, the caudex significantly wider, the flowers produced significantly more pollen, and pollen viability was higher compared to other progeny, particularly when compared to the selfed crosses. Progeny with lower genetic diversity and higher inbreeding resulted in smaller plants, reduced pollen viability, and reduced pollen mass, with the selfed progeny producing no pollen at all during this study, suggesting inbreeding depression is being expressed in ex situ collections of *B. insignis*. Plant growth (e.g., initial biomass, shoot and root biomass) has been found to be lower in more inbred progeny (Koelewijn, 1998; Pinc et al., 2020), and other studies have clearly demonstrated that inbreeding adversely affects traits related to plant male function, such as pollen production (Willis, 1993; Carr and Dudash, 1996; Good‐Avila et al., 2003; Hayes et al., 2005) and viability (Willis, 1993; Carr and Dudash, 1997). Our findings are consistent with other studies that have shown increased inbreeding coefficients among lines of plants resulted in a significant reduction in pollen viability, and many plants from the most inbred lines (*F* = 0.5 or 0.75) not producing any pollen at all (Willis, 1993). Good‐Avila et al. (2003) also found that reproductive output, including pollen production, declines with increased inbreeding in a different species of Campanulaceae (*Campanula rapunculoides* L.) and found that two generations of selfing decreased pollen production per flower by 63%.

Although *B. insignis* is primarily outcrossing, it appears to employ a mixed‐mating strategy and is capable of selfing (Walsh et al., 2019). Like all other Hawaiian lobeliads, *B. insignis* is protandrous (Lammers, 1989, 1999). The flowers start out in a male phase before entering a female phase when the style elongates through the staminal column and the stigma lobes expand (Walsh et al., 2019). It is likely that in collections not maintained by controlled pollination there is an inadvertent selection for selfing through propagation of voluntary fruit. A shift to selfing was supported by the unintentional selfed progeny resulting from the NTBG cross. Genetic diversity and percent polymorphic loci in the NTBG crossed progeny showed no genetic relatedness to the NTBG accessions and were comparable to the progeny resulting from self‐pollination. Many attempts had been made to collect pollen from all flowering plants at NTBG for inter‐institutional crosses between 2015 and 2021, with continued observations of low pollen production in plants, which was first noted as early as 2013 (Walsh et al., 2019). The very minimal amount of pollen that was collected from NTBG for the cross conducted here was a suboptimal amount and likely non‐viable. Although the NTBG progeny did produce pollen, unlike the self progeny that did not, this only occurred early in the season at the time measurements were taken. Later in the season, the flowers of NTBG progeny stopped producing pollen before progeny from the other crosses.

Unfortunately, many institutions perpetuate their living collections of exceptional species through multiple generations by collecting spontaneously formed fruits, which are likely the result of self‐pollination. When crosses are conducted, they are often done within single institutions, thus typically between genetically related accessions or different plants within the same accession (Wood et al., 2020). This is the case for many of the ex situ populations of *B. insignis*, including some of the accessions maintained in the largest, primary collection at NTBG. Using molecular tools, we were able to confirm that selfing decreases genetic diversity and increases inbreeding in *B. insignis*, but by conducting strategic crosses between collections, genetic diversity in progeny can be increased, resulting in more vigorous plants and improving the health of the collection. Performing intentional crosses with known and documented parentage can improve our ability to use pedigree information alone to guide crosses between collections. Not having to rely on molecular tools to guide strategic crosses will save significant time and resources. Pollen can be shared between institutions and likely stored long term to facilitate crosses among accessions that may not be flowering at the same time. The preliminary results of *B. insignis* pollen desiccation tolerance show that pollen is tolerant to drying at up to 5% relative humidity and cooling at −80°C for up to at least 52 weeks (J. A. Foster, unpublished data). Research is also currently underway to test the ability of *B. insignis* pollen and seeds to survive storage in liquid nitrogen, to explore their potential for long‐term storage via cryopreservation. Cryopreservation of endangered, exceptional Hawaiian species is in the early stages, with at least 14 species recently focused on for development of cryopreservation protocols and banking (Philpott et al., 2022).

Although we demonstrated that pollen production and viability decreased in progeny with decreased genetic diversity and increased inbreeding, it is also possible that low pollen production and viability in some *B. insignis* collections is due to the conditions in which plants are grown (Walsh et al., 2019). Research has shown that different environmental variables affect pollen development across a wide range of species (Lau and Stephenson, 1993; Astiz and Hernandez, 2013; Mercuri et al., 2013; Donders et al., 2014; Flores‐Rentería et al., 2018). It is also possible that a particular *B. insignis* collection could be expressing more plants that are functionally female. Field observations of wild plants from several decades ago revealed that some individuals appeared to be either gynomonoecious or monoecious (Gemmill et al., 1998). A study using *B. insignis* plants from the same, known cross type to investigate whether ex situ growing conditions (e.g., temperature, relative humidity, and nutrient availability) affect pollen production and viability would be interesting and warranted to further help improve ex situ conservation for this and other exceptional species.

We demonstrated the value of adapting a pedigree‐based approach as used by the zoo community to manage metacollections of exceptional plant species such as *B. insignis*. As many institutions maintain *B. insignis* in their collections, improving ex situ conservation for this species will require a collaboration among many institutions, which will result in increased capacity for improved ex situ conservation of other threatened species. A studbook database for plants is currently being integrated into Botanic Gardens Conservation International's (BGCI) PlantSearch, and the PMx program is being further adapted and improved for use with plants to allow curators of *B. insignis* and other exceptional species to utilize them to improve the health of their collections. There are challenges with using PMx with plants, however, as it was developed for zoos, and plant breeding is more complex than that of animals. Some obstacles include, for example, the date of birth (e.g., day seed was collected vs. day seed was sown vs. day seedling emerged) and the complexity of plant breeding systems (e.g., hermaphroditic, monoecious, dioecious, gynodioecious, androdioecious) and sexual organ expression (male, female, herkogamous, dichogamous).

Implementing a pedigree‐based approach to the management and reproduction of exceptional plant species in collections will ensure a higher level of genetic diversity and, in turn, healthier collections with a high conservation value for in situ population augmentations and reintroductions.

## AUTHOR CONTRIBUTIONS

All authors conceptualized and had a role in designing the study. A.T.K. performed the crosses, J.A.F. and J.B.F. collected molecular data, J.A.F. conducted the plant fitness measurements, and J.A.F. and J.B.F. performed statistical analyses. S.K.W. wrote the first draft of the manuscript. All authors reviewed, revised, and gave their approval for publication of the final version of the manuscript.

## Data Availability

All genetic data and plant fitness measurements of the *Brighamia insignis* accessions used in this study are freely available on Dryad (https://doi.org/10.5061/dryad.k98sf7m72; Fant et al., 2022).
